# Efficacy and safety of trimethoprim-sulfamethoxazole for the prevention of pneumocystis pneumonia in human immunodeficiency virus-negative immunodeficient patients: A systematic review and meta-analysis

**DOI:** 10.1371/journal.pone.0248524

**Published:** 2021-03-25

**Authors:** Rui Li, Zhiyong Tang, Fu Liu, Ming Yang

**Affiliations:** 1 Department of Pharmacy, The Affiliated Hospital of North Sichuan Medical College, Sichuan, Nanchong, China; 2 Department of Pharmacy, Nanchong Central Hospital, The Second Affiliated Clinical Medical College of North Sichuan Medical College, Sichuan, Nanchong, China; 3 Nanchong Key Laboratory of Individualized Drug Therapy, Sichuan, Nanchong, China; Augusta University, UNITED STATES

## Abstract

**Background:**

Pneumocystis pneumonia (PCP) has a significant impact on the mortality of immunocompromised patients. It is not known whether the prophylactic application of trimethoprim-sulfamethoxazole (TMP-SMZ) can reduce the incidence of PCP and mortality in the human immunodeficiency virus (HIV)-negative immunodeficient population. The safety profile is also unknown. There have been few reports on this topic. The aim of this study was to systematically evaluate the efficacy and safety of the use of TMP-SMZ for the prevention of PCP in this population of patients from the perspective of evidence-based medicine.

**Methods:**

A comprehensive search without restrictions on publication status or other parameters was conducted. Clinical randomized controlled trials (RCTs) or case-control trials (CCSs) of TMP-SMZ used for the prevention of PCP in HIV-negative immunocompromised populations were considered eligible. A meta-analysis was performed using the Mantel-Haenszel fixed-effects model or Mantel-Haenszel random-effects model, and odds ratios (ORs) with 95% confidence intervals (CIs) were calculated and reported.

**Results:**

Of the 2392 records identified, 19 studies (n = 4135 patients) were included. The efficacy analysis results indicated that the PCP incidence was lower in the TMP-SMZ group than in the control group (OR = 0.27, 95% CI (0.10, 0.77), p = 0.01); however, the rate of drug discontinuation was higher in the TMP-SMZ group than in the control group (OR = 14.31, 95% CI (4.78, 42.91), p<0.00001). In addition, there was no statistically significant difference in the rate of mortality between the two groups (OR = 0.54, 95% CI (0.21, 1.37), p = 0.19). The safety analysis results showed that the rate of adverse events (AEs) was higher in the TMP-SMZ group than in the control group (OR = 1.92, 95% CI (1.06, 3.47), p = 0.03).

**Conclusions:**

TMP-SMZ has a better effect than other drugs or the placebo with regard to preventing PCP in HIV-negative immunocompromised individuals, but it may not necessarily reduce the rate of mortality, the rate of drug discontinuation or AEs. Due to the limitations of the research methodologies used, additional large-scale clinical trials and well-designed research studies are needed to identify more effective therapies for the prevention of PCP.

## Introduction

*Pneumocystis jirovecii* was originally believed to be a protozoan. However, Stringer later discovered that it is an atypical fungus taxonomically located between Ascomycota and Basidiomycota that is resistant to most antifungal drugs [1]. *P*. *jirovecii* in the lungs can cause pneumocystis pneumonia (PCP), which is a severe and potentially fatal disease [2]. PCP has a significant impact on the mortality of immunocompromised patients, especially those with acquired immune deficiency syndrome (AIDS). PCP is the most common opportunistic infection and is the primary complication and cause of death in AIDS patients [3]. Because the clinical manifestations of PCP, such as shortness of breath, hypoxia, tachycardia, etc., are often the first symptoms in AIDS patients, PCP has been regarded as a hallmark disease signaling human immunodeficiency virus (HIV) infection. Consequently, PCP has received a great deal of attention. In recent years, the incidence of PCP and PCP-related mortality in HIV-positive patients have gradually decreased due to increasingly mature diagnostic technology, enabling an early diagnosis; refined intensive care management; and active prevention and treatment measures [3]. However, compared to those in HIV-positive patients, the early clinical symptoms of PCP in HIV-negative immunocompromised patients are not obvious, and delayed diagnosis may eventually lead to death due to sudden respiratory failure. Therefore, the mortality rate due to PCP in HIV-negative immunocompromised patients is higher than that in HIV-positive immunocompromised patients [4, 5]. Consequently, it is particularly important for HIV-negative immunocompromised patients to use antibacterial drugs to prevent the occurrence of PCP.

Trimethoprim-sulfamethoxazole (TMP-SMZ), the preferred preventive application, has been shown to significantly reduce the incidence of PCP in AIDS patients, and its clinical application at home and abroad is relatively mature. However, it is not known whether the prophylactic application of TMP-SMZ can reduce the incidence of PCP and PCP-related mortality in an HIV-negative immunodeficient population or what the safety profile is in that population. There have been few reports on this subject. A retrospective analysis showed that patients with rheumatic diseases who used high-dose glucocorticoids for a long time should also use sulfonamide drugs to prevent the occurrence of PCP and that the PCP incidence and mortality rates were lower in the TMP-SMZ group than in the control group [6]. In addition, some other studies indicated that up to 40% of patients with acute lymphoblastic leukemia or lymphoproliferative disease can develop PCP if they do not take prophylactic medications, and approximately 50% of PCP patients experience acute lung injury. The preventive use of TMP-SMZ in HIV-negative immunodeficient patients was found to significantly reduce the incidence of adverse events (AEs) in these patients compared with the control group (33:1) [7, 8]. Furthermore, patients with immunodeficiency due to causes other than infection with HIV, such as those undergoing allogeneic stem cell transplantation or solid organ transplantation and those with multiple myeloma, are all recommended to take TMP-SMZ to prevent PCP [9–11]. However, there is still a lack of clinical evidence regarding the effectiveness and safety of the prophylactic use of TMP-SMZ in this population.

To address this knowledge gap, we performed a systematic analysis of the efficacy and safety of the prophylactic use of TMP-SMZ to prevent PCP in HIV-negative immunocompromised patients from the perspective of evidence-based medicine to provide a reference for clinical decision-making and promote the rational use of drugs in clinical practice.

## Methods

Our study protocol and analysis were planned in accordance with the Preferred Reporting Items for Systematic Reviews and Meta-Analyses (PRISMA) guidelines, Parts of the methodology can be found in the article by Rui Li et al. [12].

### Search strategy

A systematic search of the following electronic databases was performed to identify relevant literature published in English before December 24, 2020: PubMed, EMBASE, Web of Science, and the Cochrane Library. The search strategy included the following medical index terms: “pneumocystis pneumonia,” “pneumocystis infections,” “pneumocystis jirovecii,” “prophylactic,” “prophylaxis,” “prevention,” “trimethoprim sulfamethoxazole,” “HIV,” “AIDS,” “acquired immunodeficiency syndrome,” and “human immunodeficiency virus”. Furthermore, the references in the initially identified articles, including relevant reviews, were manually searched and reviewed to ensure that no relevant study was missed (up to December 24, 2020).

### Study selection

Studies were included if they met the following inclusion criteria: (1) they were randomized controlled trials (RCTs) or case-control studies (CCSs), (2) they contained data regarding the preventative use of TMP-SMZ by HIV-negative immunodeficient patients, and (3) they contained data on the incidence of PCP, the rate of drug discontinuation (discontinuation due to AEs or patient intolerance), the rate of mortality or the rate of AEs. Studies were excluded if they (1) were unrelated to PCP, (2) were duplicate reports, (3) had irrelevant data, or (4) were not RCTs or CCSs.

All retrieved studies were scanned by two reviewers (RL and ZYT), who independently assessed all potentially relevant studies and then reached a consensus. Then, relevant studies were examined to obtain data on the incidence of PCP and the rates of drug discontinuation, mortality and AEs. In the case of disagreement between the two reviewers, the senior coauthor (MY) was consulted, and the disagreement was resolved by consensus.

### Data extraction

Two reviewers (RL and ZYT) extracted the relevant data from each eligible study independently, and discrepancies were resolved through discussion with the senior coauthor (MY). A predefined form was used to record the following information: (1) first author; (2) year of publication; (3) research type; (4) mean age; (5) proportion of males; (6) follow-up period; (7) numbers of patients in the treatment group and the control group; (8) therapeutic regimen; (9) incidence rate of PCP; (10) rate of drug discontinuation; (11) mortality rate; (12) AEs related to the study medications; (13) overall risk of bias; and (14) quality of the evidence.

### Quality appraisal and assessment of the risk of bias

The quality of each included study was evaluated according to the modified Jadad score [13]. Two reviewers (RL and ZYT) independently assessed the methodological quality of all included studies without blinding regarding the source journal or authorship. Disagreements were resolved by discussion or consultation with the third reviewer (MY) if required. The risk of bias in each included study was also evaluated [14]. Potential publication bias was assessed by the visual inspection of asymmetry in Begg’s funnel plots, and Egger’s test was then used to provide statistical evidence of funnel plot symmetry (p<0.05 indicating bias and p>0.05 indicating no bias) [15, 16].

### Data analysis and statistical methods

All statistical analyses, except for the publication bias analysis (which was performed using STATA software: version 12.0, StataCorp, College Station, TX, USA), were performed using Review Manager software (RevMan, version 5.1, Oxford, UK; The Cochrane Collaboration, 2008). The heterogeneity among studies was initially assessed graphically by examining forest plots and was subsequently assessed statistically with the chi-square test for homogeneity, and both I^2^ statistics and p-values were considered [17]. A p-value <0.1 or I^2^>50% indicated high heterogeneity among studies. An I^2^ value between 25% and 50% indicated moderate heterogeneity, and a p-value >0.1 or I^2^<25% signified low heterogeneity [14]. Pooled odds ratios (ORs; calculated by adding 0.5 to each cell of the 2×2 table for the trial when one arm of the study contained no events [18]) and 95% confidence intervals (CIs) were also used in the meta-analysis. The meta-analysis was performed using the Mantel-Haenszel fixed-effects model (FEM) or Mantel-Haenszel random-effects model (REM).

### Outcomes analyzed

In this meta-analysis, regarding the outcome measures used to assess efficacy, the incidence of PCP was used as the primary outcome measure, and the rate of drug discontinuation and the rate of mortality were used as the secondary outcome measures. We performed subgroup analyses according to the therapeutic intervention, e.g., TMP-SMZ prevention group vs nonprevention group and TMP-SMZ vs other drugs. The rate of AEs was used as the primary safety outcome measure in this meta-analysis. We compared the TMP-SMZ group with the control group. According to the clinical manifestations of AEs (i.e., rash, hematologic system effects, infection, abnormal liver and renal function and other AEs), we divided the data into five subgroups for analysis and comparison.

## Results

### Search results

**Fig 1:**
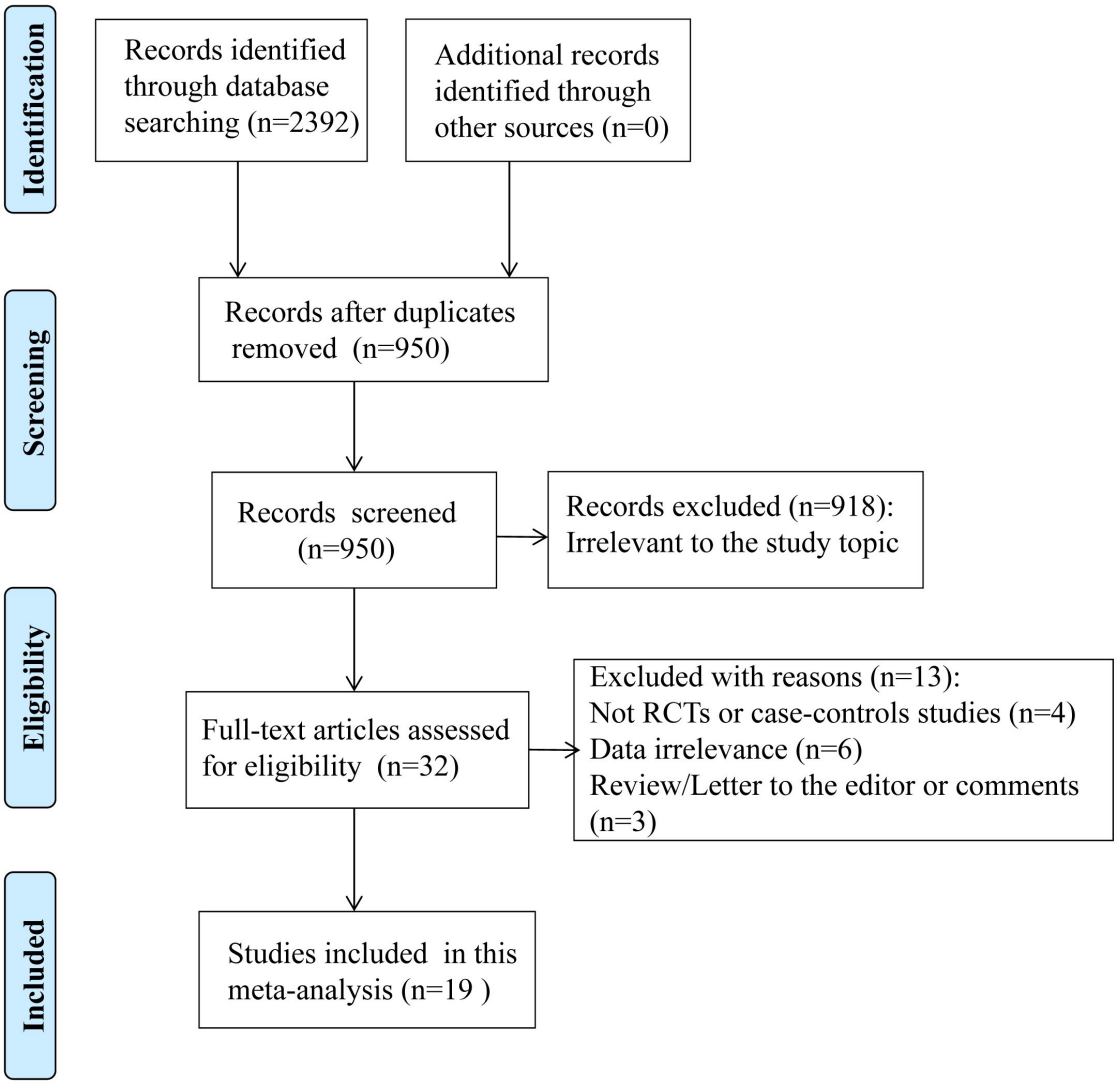
Flow diagram of studies included in the meta-analysis. PRISMA flow diagram showing the number of articles identified and evaluated during the review.

The complete search strategy used for each database is described in S1 Table. The detailed process of the literature search and article screening process is described in Fig 1. A total of 2392 records were identified from the English databases, and 0 records were identified through other searches. After excluding duplicates and screening the titles of the studies, 950 articles remained for further review. An additional 918 articles were excluded after reading the abstracts of the potentially relevant articles, and 32 were subjected to a full-text review based on their relevance to the study topic. Finally, a total of 19 articles with 4135 patients were included after the exclusion of 13 articles that were not RCTs or CCSs, did not report the outcomes of interest or were reviews, letters to the editor or comments [6, 10, 19–32].

### Study quality assessment and risk of bias assessment

The quality of each included study was assessed with the modified Jadad score (S2 Table). The results indicated that five studies were of high quality [23–25, 34, 35]. The majority of the studies (fourteen studies) were of moderate quality (Table 1, S2 Table) [6, 10, 19–22, 26–33]. The results of the assessment of the overall risk of bias for each included study indicated that five reports exhibited a low risk of bias [23–25, 34, 35] and that the remaining fourteen reports exhibited an unclear risk of bias (Table 1, S1 Fig) [6, 10, 19–22, 26–33]. The summaries and characteristics of the included articles are presented in Table 1.

**Table 1:** Main characteristics of the studies included in the meta-analysis.

Study	Research Type	Mean Age	Male (%)	Follow-up (months)	Enrolled Patients		Drug Regimen		Assessment Index	Evidence Quality	Risk of Bias
					T	C	T	C			
**TMP-SMZ vs. Nonprevention**											
Park, 2017 [6]	CCS	43.7	22	12	262	1260	TMP-SMZ	Nonprevention	(1)(3)	Moderate	Unclear
Neofytos, 2018 [10]	CCS	65	36	12	41	2801	TMP-SMZ	Nonprevention	-1	Moderate	Unclear
Katsuyama, 2014 [19]	CCS	58.5	19	16	141	561	TMP-SMZ	Nonprevention	(1)(4)	Moderate	Unclear
Ogawa, 2005 [20]	CCS	56.2	52	6	49	75	TMP-SMZ	Nonprevention	(1)(4)	Moderate	Unclear
Okada, 1999 [21]	CCS	38	21	2.5	37	47	TMP-SMZ	Nonprevention	(1)(4)	Moderate	Unclear
Colby,1999 [22]	RCT	45.5	28	3	18	16	TMP-SMZ	Nonprevention	(2)(4)	Moderate	Unclear
Vananuvat, 2011 [23]	RCT	34.5	91	3	59	79	TMP-SMZ	Nonprevention	(1)(4)	High	Low
Levinsen, 2011 [24]	RCT	4	43	12	112	250	TMP-SMZ	Nonprevention	(1)(3)	High	Low
Ward, 1993 [25]	RCT	55.4	92.9	1	22	20	TMP-SMZ	Nonprevention	(1)(3)	High	Low
**TMP-SMZ vs. Other drugs**											
Evans, 2015 [26]	CCS	56.2	58	12	79	79	TMP-SMZ	Dapsone	-4	Moderate	Unclear
Nazir, 2017 [27]	CCS	7	55	12	24	34	TMP-SMZ	Dapsone	(1)(2)(4)	Moderate	Unclear
Schmajuk, 2018 [28]	CCS	43	20	6	129	28	TMP-SMZ	Dapsone	-4	Moderate	Unclear
Redjoul, 2018 [29]	CCS	56	60	12	113	13	TMP-SMZ	Atovaquone	(1)(2)(3)(4)	Moderate	Unclear
Gabardi, 2012 [30]	CCS	52	64	12	160	25	TMP-SMZ	Atovaquone	(2)(4)	Moderate	Unclear
Zmarlicka, 2015 [31]	CCS	50.7	63	12	67	11	TMP-SMZ	Atovaquone	(3)(4)	Moderate	Unclear
Kimura, 2008 [32]	CCS	49.4	33	12	27	19	TMP-SMZ	Pentamidine	(1)(4)	Moderate	Unclear
Sangiolo, 2005 [33]	CCS	44	42	6	310	155	TMP-SMZ	Dapsone	-1	Moderate	Unclear
Kitazawa, 2019 [34]	CCS	66.5	22	12	55	28	TMP-SMZ	Pentamidine	(2)(4)	High	Low
Hughes, 1977 [35]	RCT	<18	Unclear	Unclear	80	80	TMP-SMZ	Atovaquone	-1	High	Low

Characteristics of the included RCTs or CCSs comparing TMP-SMZ with a placebo or other drugs for the prevention of PCP in HIV-negative immunocompromised patients.

Abbreviations: CCS: case-control study; RCT: randomized controlled trial; T: treatment (TMP-SMZ); C: control (nonprevention or other drugs); (1): PCP incidence; (2): the rate of drug discontinuation; (3): mortality; (4): AEs.

### Efficacy outcomes

#### PCP incidence

**Fig 2:**
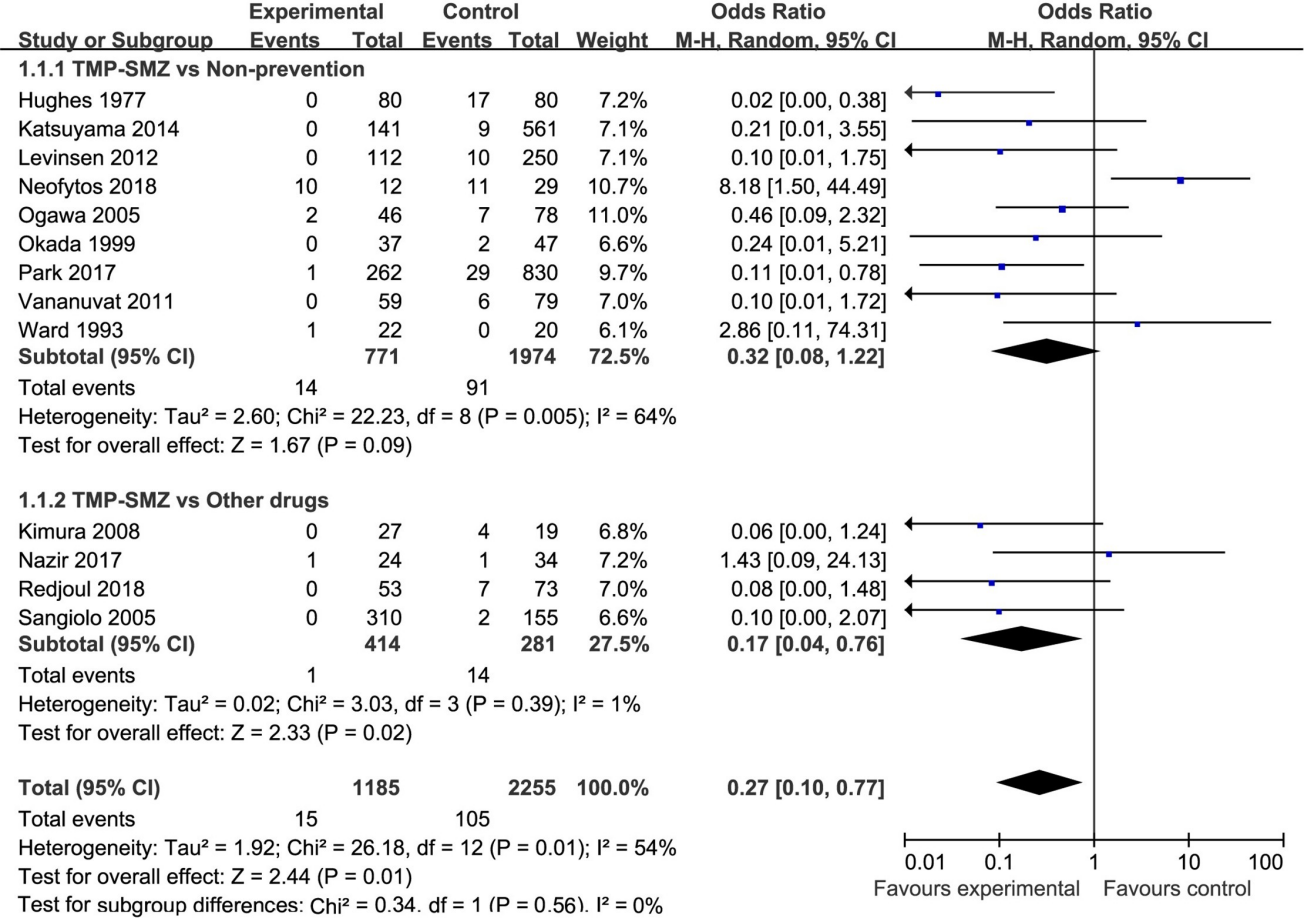
Forest plot of the incidence rate of PCP incidence. The vertical line indicates no difference between the groups. ORs are represented by diamonds, and 95% CIs are depicted by horizontal lines. Squares indicate point estimates, and the size of each square indicates the weight of the given study in the meta-analysis. M-H, Mantel-Haenszel random-effects model.

There were thirteen studies concerning the PCP incidence [6, 10, 19–21, 23–25, 27, 29, 32, 33, 35]. We categorized them into two subgroups: TMP-SMZ prevention vs. nonprevention and TMP-SMZ vs. other drugs (that prevent PCP infection), for statistical analysis (Fig 2). A total of 1185 patients were included in the TMP-SMZ group, and 2255 patients were included in the control group (nonprevention or other drugs). The meta-analysis results suggested that there was significant heterogeneity among these studies (p = 0.01, I^2^ = 54%). With regard to comparison between the TMP-SMZ group and the control group, the results of the meta-analysis showed a significant difference in the incidence of PCP (OR = 0.27, 95% CI (0.10, 0.77), p = 0.01), and the incidence of PCP in the TMP-SMZ group was significantly lower than that in the control group (Fig 2).

#### Rate of drug discontinuation

**Fig 3:**
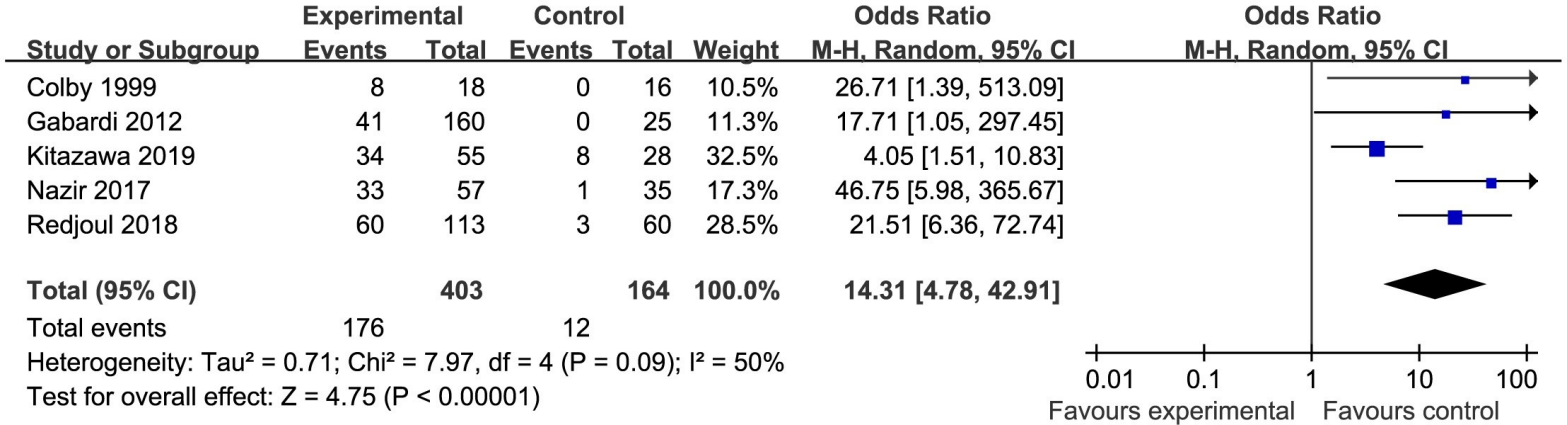
Forest plot of the rate of drug discontinuation. The vertical line indicates no difference between the groups. ORs are represented by diamonds, and 95% CIs are depicted by horizontal lines. Squares indicate point estimates, and the size of each square indicates the weight of the given study in the meta-analysis. M-H, Mantel-Haenszel random-effects model.

Five studies were included in the rate of drug discontinuation analysis [22, 27, 29, 30, 34]. A total of 403 patients were included in the TMP-SMZ group, and 164 patients were included in the control group (other drugs to prevent PCP). The meta-analysis results indicated that there was significant heterogeneity among these studies (p = 0.09, I^2^ = 50%). With regard to the comparison between the TMP-SMZ group and the control group, the results of the meta-analysis showed a significant difference in the drug discontinuation rate (OR = 14.31, 95% CI (4.78, 42.91, p<0.00001); the rate of drug discontinuation in the TMP-SMZ group was significantly higher than that in the control group (Fig 3).

#### Rate of mortality

**Fig 4:**
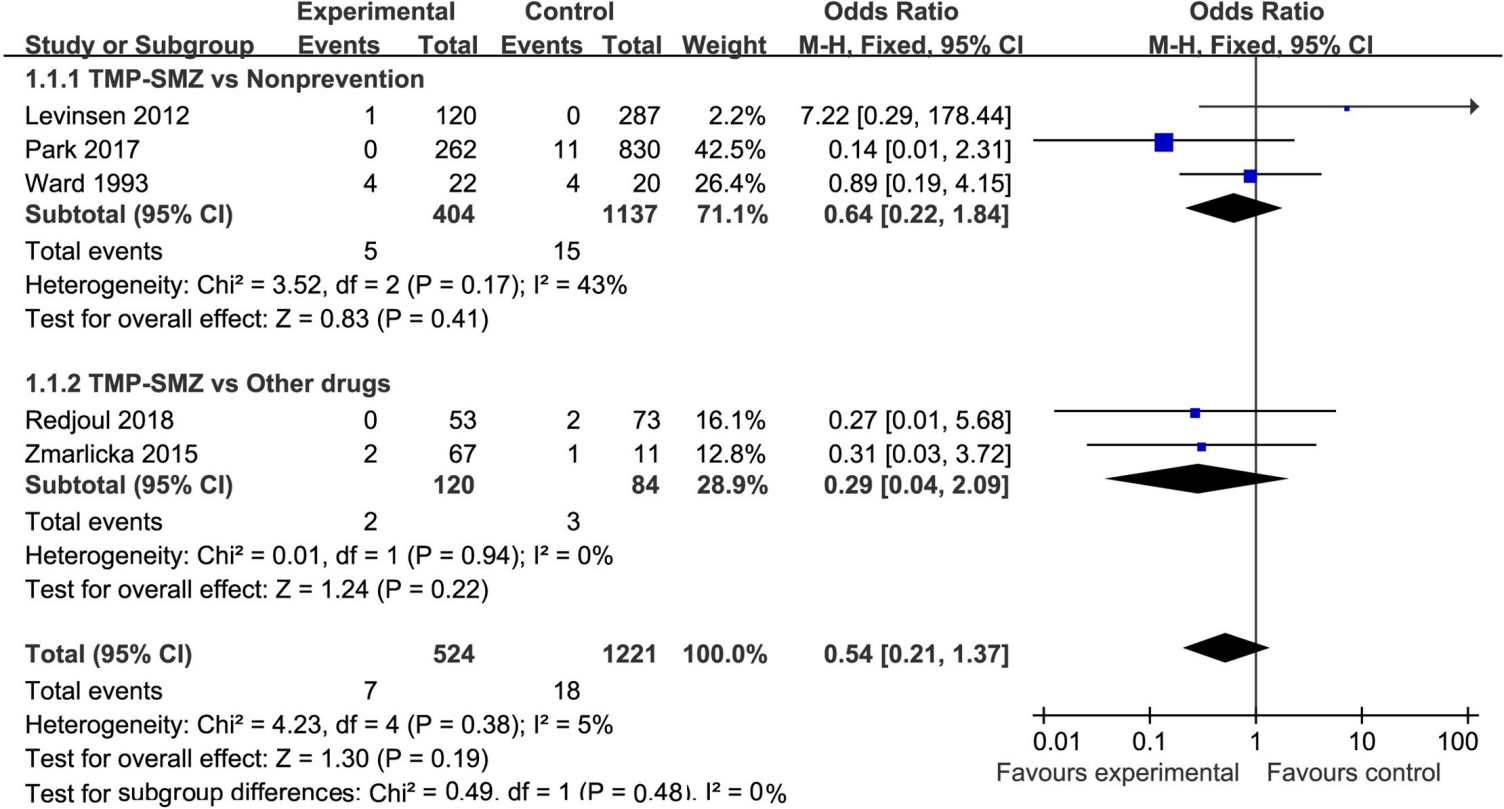
Forest plot of the rate of mortality. The vertical line indicates no difference between the groups. ORs are represented by diamonds, and 95% CIs are depicted by horizontal lines. Squares indicate point estimates, and the size of each square indicates the weight of the given study in the meta-analysis. M-H, Mantel-Haenszel fixed-effects model.

In the analysis of the rate of mortality, five studies were included [6, 24, 25, 29, 31], which were categorized into two subgroups (TMP-SMZ vs. nonprevention and TMP-SMZ vs. other drugs to prevent PCP) (Fig 4). A total of 524 patients were included in the TMP-SMZ group, and 1221 patients were included in the control group (nonprevention or other drugs). The meta-analysis results did not show significant heterogeneity among these studies (I^2^ = 5%, p = 0.38). With regard to the comparison of the TMP-SMZ group and the control group, there was no statistically significant difference in the rate of mortality between the two groups (OR = 0.54, 95% CI (0.21, 1.37), p = 0.19) (Fig 4).

### Safety outcomes

#### Rate of AEs

**Fig 5:**
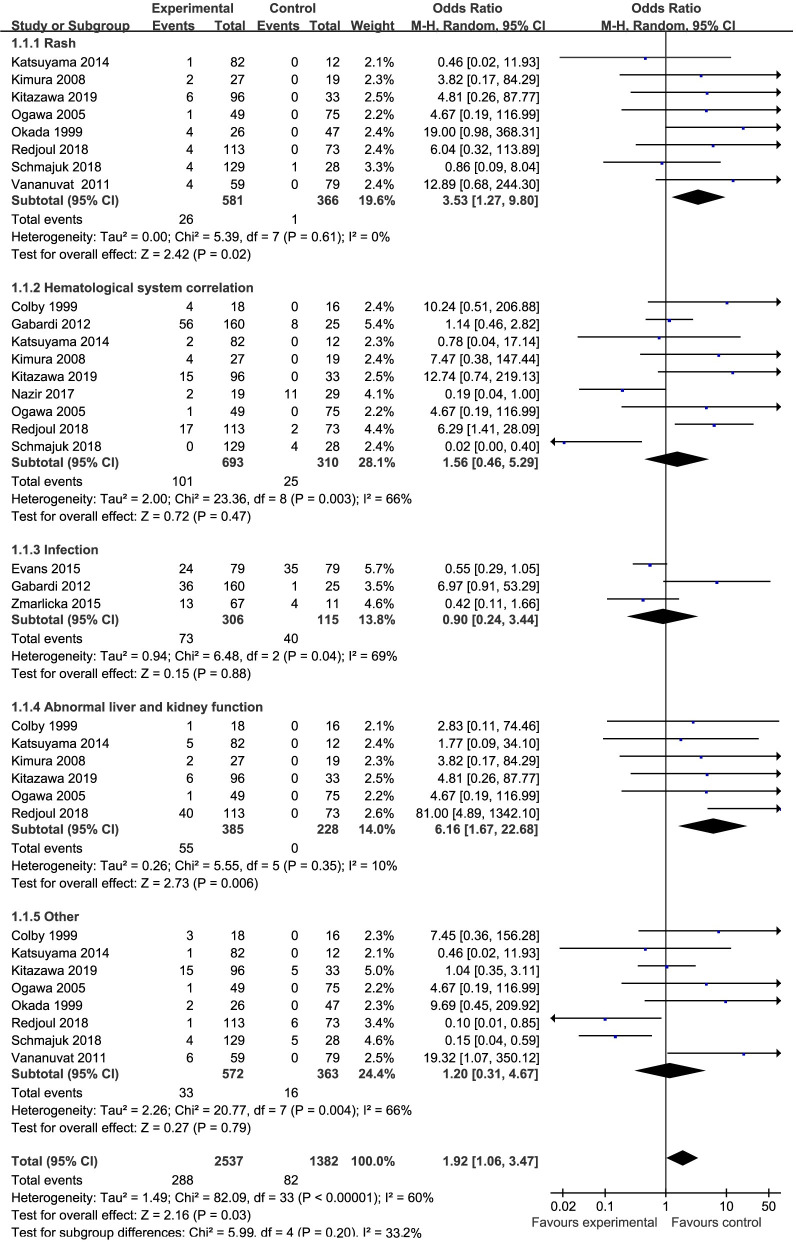
Forest plot of the rate of AEs. The vertical line indicates no difference between the groups. ORs are represented by diamonds, and 95% CIs are depicted by horizontal lines. Squares indicate point estimates, and the size of each square indicates the weight of the given study in the meta-analysis. M-H, Mantel-Haenszel random- effects model.

The reported AEs in the included studies were rash, hematologic system effects, infections, liver and kidney dysfunction, and other AEs [19–23, 26–32, 34]. We performed subgroup analyses for each category of AEs. The meta-analysis results indicated that there was significant heterogeneity among these studies (I^2^ = 60%, p<0.00001) and that the rate of AEs was higher in the TMP-SMZ group than in the control group. The results from thirteen studies were separated into five subgroups (OR = 1.92, 95% CI (1.06, 3.47), p = 0.03) (Fig 5).

### Meta-analysis stratified by study design

Most of the included articles were CCSs. Therefore, to verify whether the study design had a significant impact on the outcome measures, we also conducted meta-analyses of the RCTs and CCSs separately for each outcome measure. Because only one RCT reported the rate of drug discontinuation, no comparative analysis was performed. The results of the meta-analysis showed that the incidence of PCP (p = 0.05 vs. p = 0.01) and the incidence of AEs (p = 0.0006 vs. p = 0.03) were statistically significant, while the mortality rate was not statistically significant (p = 0.63 vs. p = 0.19) when comparing RCTs with RCTs and CCSs (the efficacy and safety outcomes above). The summary data of the meta-analysis for each outcome measure stratified by study design are presented in Table 2.

**Table 2:** Summary of the meta-analysis of each outcome measure stratified by study design.

Outcome Measure	Research Type	Included Studies	OR	95% CI	p
PCP incidence	RCTs	4	0.14	[0.02, 0.97]	0.05*
	CCSs	9	0.36	[0.10, 1.20]	0.10
	RCTs+CCSs	13	0.27	[0.10, 0.77]	0.01
Rate of mortality	RCTs	2	1.39	[0.37, 5.22]	0.63
	CCSs	3	0.20	[0.04, 1.03]	0.05*
	RCTs+CCSs	5	0.54	[0.21, 1.37]	0.19
Rate of AEs	RCTs	2	10.11	[2.69, 38.02]	0.0006
	CCSs	11	1.55	[0.83, 2.88]	0.17
	RCTs+CCSs	13	1.92	[1.06, 3.47]	0.03

Abbreviations: PCP: pneumocystis pneumonia; AEs: adverse events; RCTs: randomised controlled trials; CCSs: case-control studies; OR: odds ratio; CI: confidence interval.

*: marginal p-value, there was a significant difference, but further analysis is needed with a larger sample size.

### Publication bias and sensitivity analyses

In this article, we performed publication bias analyses for the incidence of PCP, rate of drug discontinuation and rate of mortality, and Begg’s funnel plots were drawn using STATA 12.0 software. In S2 Fig, each small circle represents a study. Visual inspection of the three funnel plots showed that the majority of small circles were roughly symmetrically distributed above, below and to the left and right on the Begg’s funnel diagram, except for the individual small circles distributed outside the funnel diagram, which may be due to the significant heterogeneity among these studies. Egger’s test was performed for each outcome, and the results are shown in S3 Table: all p-values were greater than 0.05, which further proved that the above three funnel plots were symmetrical, and the individual small outliers may be due to heterogeneity among these studies rather than to publication bias. Therefore, both the shape of Begg’s funnel plot and Egger’s test results (all p>0.05) demonstrate that there was no publication bias in the included studies.

The meta-analysis showed significant heterogeneity among the studies for the incidence of PCP (I^2^ = 54%) and the rate of drug discontinuation (I^2^ = 50%). On the one hand, age, follow-up duration and research type may have had substantial impacts on these two indicators. Thus, we conducted sensitivity analyses with stratification by these two indicators to explore the underlying sources of heterogeneity. The final analysis showed that the primary results were not influenced by age, follow-up duration or research type. The detailed data are shown in S4 Table. On the other hand, the included studies were excluded one by one for each outcome indicator (the incidence of PCP and the rate of drug discontinuation) to analyze the sources of heterogeneity. The results are shown in S5 Table. The heterogeneity among the studies reporting the incidence of PCP was mainly due to the study by Neofytos, and the heterogeneity among the studies reporting the drug discontinuation rate was mainly due to the study by Kitazawa [10, 34].

## Discussion

The aim of this study was to systematically evaluate the efficacy and safety of the prophylactic application of TMP-SMZ to prevent PCP in HIV-negative immunocompromised patients from the perspective of evidence-based medicine. This study has revealed that TMP-SMZ has a better effect than other drugs or placebo on preventing PCP in HIV-negative immunocompromised people. The prophylactic use of TMP-SMZ compared to a placebo or other drugs can significantly reduce the incidence of PCP in such patients, resulting in a lower rate of mortality. However, the rate of drug discontinuation for TMP-SMZ was significantly higher than that for other drugs. Moreover, the meta-analysis showed that the rate of AEs was also higher in the TMP-SMZ group than in the control group.

Although RCTs generally produce higher-quality evidence than CCSs because they eliminate selection bias, the Cochrane handbook also states that consolidation is acceptable when observational studies have large sample sizes or are of high quality or when RCTs have a small sample size or mediocre quality [14]. Considering the small total number of RCTs and the meta-analysis indicated that there was no significant impact of the study design on the outcome measures. Therefore, in the results section, we present the meta-analysis of the RCTs and CCSs combined.

Regarding efficacy, the incidence of PCP in the TMP-SMZ group was significantly lower than that in the control group, but the rate of discontinuation of TMP-SMZ was significantly higher than that in the control group. While there was no statistically significant difference in the rate of mortality between the two groups, the mortality in the TMP-SMZ group was lower than that in the control group. The Cochrane meta-analysis showed that TMP-SMZ prophylaxis resulted in a 91% reduction in the incidence of PCP and an 83% reduction in the rate of mortality compared to the control group [7], which was similar to the results reported in this study. Although TMP-SMZ is the first choice for the prevention of PCP, TMP-SMZ often has to be replaced with second-line drugs such as dapsone, atomized pentamidine, and atovaquone due to AEs associated with TMP-SMZ [7, 27, 30], and drug intolerance caused by G6PD deficiency and neutropenia. Therefore, the rate of discontinuation of TMP-SMZ was significantly higher than that in the control group. Regarding safety, the meta-analysis showed that the rate of AEs was higher in the TMP-SMZ group than in the control group. Although the TMP-SMZ group had fewer cases of secondary infections than the control group (23.86% vs. 34.78%; Fig 5), the number of AEs was higher in the TMP-SMZ group than in the control group, including rash, hematologic system effects (including methemoglobinemia, thrombocytopenia and agranulocytosis, etc.), liver and kidney dysfunction and other AEs (including asthma, nausea, vomiting, hyponatremia, etc.). Most of these AEs can be eliminated or improved after drug withdrawal, but the AEs associated with the blood system (e.g., agranulocytosis) can lead to serious opportunistic infections accompanied by fever in these immunocompromised patients. Hence, appropriate drugs should be selected for inclusion in the regimen according to the type and severity of the patient’s disease, and the occurrence of AEs should be closely monitored.

The sensitivity analysis suggested that age, follow-up duration and research type may not be sources of heterogeneity. We excluded the included studies one by one from the main outcome analysis and found that the heterogeneity was primarily due to the clinical trial methodology in the included literature, such as in the studies by Neofytos and Kitazawa [10, 34]. The diagnosis of PCP in the study by Neofytos was entirely based on polymerase chain reaction (PCR), and one of the main limitations of that study was that the PCR cycle threshold could not be used to diagnose PCR-positive cases. Although only 4 cases were diagnosed based on a positive PCR for Pneumocystis in that study, it ultimately affected the diagnosis and prevention of PCP. In the study by Kitazawa, there were significant differences in the baseline characteristics of the population using the prophylaxis. Although the same drugs were used, the heterogeneity may have been caused by the differences in baseline conditions and the characteristics of the patients, and false negative or false positive results might have occurred.

Our study has several limitations. First, the nineteen studies included populations from different countries, so the results could have been affected by region, ethnicity, or language, leading to bias. Second, although the quality of the majority of the studies was moderate, the small overall sample size may have led to false positive or false negative results. Third, most of the studies were CCSs, and the method of randomization was unclear or not mentioned in most studies, indicating the possibility of selection bias. Fourth, the heterogeneity among some of the included studies could have resulted in bias. Fifth, although the results of the sensitivity analyses stratified by age, follow-up duration and research type did not show any significant effects, the large range in follow-up durations (1–12 months) may have resulted in bias.

In conclusion, these findings have important clinical implications for the prevention of PCP in HIV-negative immunocompromised people. However, due to the limitations of the research methodology, these conclusions need to be further verified in large-scale, prospective RCTs to provide reasonable guidance with regard to the prevention of PCP.

## Supporting information

S1 Checklist PRISMA checklist for the meta-analysis. (DOC)

S1 Table Search strategy. (DOC)

S2 Table Assessment of the quality of evidence in each included study with the modified JADAD score. (DOC)

S3 Table Egger’s tests for the three Begg’s funnel plots. (DOC)

S4 Table Sensitivity analyses of the incidence of PCP and rate of drug discontinuation. (DOC)

S5 Table Heterogeneity analysis for the incidence of PCP and rate of drug discontinuation. (DOC)

S1 Fig Risk of bias summary. Each risk of bias item for each included study was reviewed.
(TIF)

S2 Fig Begg’s funnel plots for the meta-analysis of the incidence of PCP (A), rate of drug discontinuation (B) and rate of mortality (C).
(TIF)

## References

[1] Stringer JR. Pneumocystis carinii: what is it, exactly? Clin Microbiol Rev. 1996;9:489–498. 10.1128/CMR.9.4.489-498.1996 8894348 PMC172906

[2] Thomas CF, Limper AH. Pneumocystis pneumonia. N Engl J Med. 2004;350:2487–2498. 10.1056/NEJMra032588 15190141

[3] Huang L, Cattamanchi A, Davis JL, den Boon S, Kovacs J, Meshnick S, et al. HIV-associated Pneumocystis pneumonia. Proc Am Thorac Soc. 2011;8:294–300. 10.1513/pats.201009-062WR 21653531 PMC3132788

[4] Catia C, Cristina D, Míriam JM, Asunción M, Felipe G, Antoni T, et al. Pneumocystis pneumonia in the twenty-first century: HIV-infected versus HIV-uninfected patients. Expert Rev Anti Infect Ther. 2019;17(10):787–801. 10.1080/14787210.2019.1671823 31550942

[5] Helmut FS, Guido S, Martin H, Gunar G, Christian H, Barbara K, et al. Clinical, Diagnostic, and Treatment Disparities between HIV-Infected and Non-HIV-Infected Immunocompromised Patients with Pneumocystis jirovecii Pneumonia. Respiration. 2018;96(1):52–65. 10.1159/000487713 29635251

[6] Park JW, Curtis JR, Moon J, Song YW, Kim S, Lee EB. Prophylactic effect of trimethoprim-sulfamethoxazole for pneumocystis pneumonia in patients with rheumatic diseases exposed to prolonged high-dose glucocorticoids. Ann Rheum Dis. 2018;77:644–9. 10.1136/annrheumdis-2017-211796 29092853 PMC5909751

[7] Hughes WT, Feldman S, Aur RJ, Verzosa MS, Hustu HO, Simone JV. Intensity of immunosuppressive therapy and the incidence of Pneumocystis carinii pneumonitis. Cancer. 1975;36:2004–9. 10.1002/cncr.2820360912 1081905

[8] Nazir HF, Elshinawy M, AlRawas A, Khater D, Zadjaly S, Wali Y. Efficacy and Safety of Dapsone Versus Trimethoprim/Sulfamethoxazol for Pneumocystis Jiroveci Prophylaxis in Children With Acute Lymphoblastic Leukemia With a Background of Ethnic Neutropenia. J Pediatr Hematol Oncol. 2017;39:203–208. 10.1097/MPH.0000000000000804 28234744

[9] Molina A, Winston DJ, Pan D, Schiller GJ. Increased Incidence of Nocardial Infections in an Era of Atovaquone Prophylaxis in Allogeneic Hematopoietic Stem Cell Transplant Recipients. Biol Blood Marrow Transplant. 2018;24:1715–1720. 10.1016/j.bbmt.2018.03.010 29555315

[10] Neofytos D, Hirzel C, Boely E, Lecompte T, Khanna N, Mueller NJ, et al. Pneumocystis jirovecii pneumonia in solid organ transplant recipients: a descriptive analysis for the Swiss Transplant Cohort. Transpl Infect Dis. 2018;20:e12984. 10.1111/tid.12984 30155950

[11] Vesole DH, Oken MM, Heckler C, Greipp PR, Katz MS, Jacobus S, et al. Oral antibiotic prophylaxis of early infection in multiple myeloma: a URCC/ECOG randomized phase III study. Leukemia. 2012;26:2517–2520. 10.1038/leu.2012.124 22678167 PMC4734137

[12] Rui L, Laichun L, Xin L, Mingxia W, Xin L, et al. Efficacy and safety of metronidazole monotherapy versus vancomycin monotherapy or combination therapy in patients with Clostridium difficile infection: A systematic review and meta-analysis. PlosOne.2015;10(7): 1–14. 10.1371/journal.pone.0137252 PMC4621873 26444424

[13] Jadad AR, Moore RA, Carroll D, Jenkinson C, Reynolds DJ, Gavaghan DJ, et al. Assessing the quality of reports of randomized clinical trials: is blinding necessary? Controlled clinical trials.1996;17:1–12. 10.1016/0197-2456(95)00134-4 8721797

[14] Higgins JP, Green S. Cochrane Handbook for Systematic Reviews of Interventions Version 5.1.0 (updated March 2011). The Cochrane Collaboration 2011. Available: http://www.cochrane-handbook.org/ [December 2014].

[15] Egger M, Davey Smith G, Schneider M, Minder C. Bias in meta-analysis detected by a simple,graphical test. Bmj. 1997;315:629–634. 10.1136/bmj.315.7109.629 9310563 PMC2127453

[16] Sterne JA, Egger M, Smith GD. Systematic reviews in health care: investigating and dealingwith publication and other biases in meta-analysis. Bmj. 2001;323:101–105. 10.1136/bmj.323.7304.101 11451790 PMC1120714

[17] Wynd S, Westaway M, Vohra S, Kawchuk G. Correction: Acetyl-L-carnitine in the Treatment of Peripheral Neuropathic Pain: A Systematic Review and Meta-analysis of Randomized Controlled Trials. PloS one. 2015;10:e0129991. 10.1371/journal.pone.0129991 26065423 PMC4466799

[18] Friedrich JO, Adhikari NK, Beyene J. Inclusion of zero total event trials in meta-analyses maintains analytic consistency and incorporates all available data. BMC medical researchMethodology. 2007;7:5. 10.1186/1471-2288-7-5 17244367 PMC1783664

[19] Katsuyama T, Saito K, Kubo S, Nawata M, Tanaka Y. Prophylaxis for Pneumocystis pneumonia in patients with rheumatoid arthritis treated with biologics, based on risk factorsfound in a retrospective study. Arthritis Res Ther. 2014;16:R43. 10.1186/ar4472 24495443 PMC3978920

[20] Ogawa J, Harigai M, Nagasaka K, Nakamura T, Miyasaka N. Prediction of and prophylaxis against Pneumocystis pneumonia in patients with connective tissue diseases undergoingmedium-or high-dose corticosteroid therapy. Mod Rheumatol. 2005;15:91–96. 10.1007/pl00021707 17029042

[21] Okada J, Kadoya A, Rana M et al. Efficacy of sulfamethoxazole-trimethoprim administration in the prevention of Pneumocystis carinii pneumonia in patients with connective tissue disease. Kansenshogaku Zasshi. 1999;73:1123–1129. 10.11150/kansenshogakuzasshi1970.73.1123 10624092

[22] Colby C, McAfee SL, Sackstein R, Finkelstein DM, Fishman JA, Spitzer TR. A prospective randomized trial comparing the toxicity and safety of atovaquone with trimethoprim/ sulfamethoxazole as Pneumocystis carinii pneumonia prophylaxis following autologousperipheral blood stem cell transplantation. Bone Marrow Transplantation. 1999;24, 897–902. 10.1038/sj.bmt.1702004 10516703

[23] Vananuvat P, Suwannalai P, Sungkanuparph S, Limsuwan T, Ngamjanyaporn P, Janwityanujit S. Primary prophylaxis for Pneumocystis jirovecii pneumonia in patients withconnective tissue diseases. Semin Arthritis Rheum. 2011;41: 497–502. 10.1016/j.semarthrit.2011.05.004 21959291

[24] Levinsen M, Shabaneh D, Bohnstedt C, Harila-Saari A, Jonsson OG, Kanerva J, et al. Pneumocystis jiroveci pneumonia prophylaxis during maintenance therapy influences methotrexate/6-mercaptopurine dosing but not event-free survival for childhood acutelymphoblastic leukemia. Eur J Haematol. 2012;88:78–86. 10.1111/j.1600-0609.2011.01695.x 21854453

[25] Ward TT, Thomas GR, Fye LC, Arbeit R, Coltman AC, Craig WJ, et al. Trimethoprim-Sulfamethoxazole Prophylaxis in Granulocytopenic Patients with Acute Leukemia: Evaluation of Serum Antibiotic Levels in a Randomized, Double-Blind, Placebo-Controlled Department ofVeterans Affairs Cooperative Study. Clinical Infectious Diseases. 1993;17:323–332 10.1093/clinids/17.3.323 8218671

[26] Evans RA, Clifford TM, Tang S, Au T, Fugit AM. Efficacy of once-weekly dapsone dosing for Pneumocystis jirovecii pneumonia prophylaxis post transplantation. Transpl Infect Dis.2015;17:816–821. 10.1111/tid.12457 26369753

[27] Nazir HF, Elshinawy M, AlRawas A, Khater D, Zadjaly S, Wali Y. Efficacy and Safety of Dapsone Versus Trimethoprim/Sulfamethoxazol for Pneumocystis Jiroveci Prophylaxis in Children With Acute Lymphoblastic Leukemia With a Background of Ethnic Neutropenia. J Pediatr Hematol Oncol. 2017;39:203–208. 10.1097/MPH.0000000000000804 28234744

[28] Schmajuk G, Jafri K, Evans M, Shiboski S, Gianfrancesco M, Izadi Z, et al. Pneumocystis jirovecii pneumonia (PJP) prophylaxis patterns among patients with rheumatic diseasesreceiving high-risk immunosuppressant drugs. Semin Arthritis Rheum. 2019;48:1087–1092. 10.1016/j.semarthrit.2018.10.018 30449650 PMC6499720

[29] Redjoul R, Robin C, Foulet F, Leclerc M, Beckerich F, Cabanne L, et al. Pneumocystis jirovecii pneumonia prophylaxis in allogeneic hematopoietic cell transplant recipients: can wealways follow the guidelines? Bone marrow transplantation. 2019;54:1082–1088. 10.1038/s41409-018-0391-2 30413810

[30] Gabardi S, Millen P, Hurwitz S, Martin S, Roberts K, Chandraker A. Atovaquone versus trimethoprim-sulfamethoxazole as pneumocystis jirovecii pneumonia prophylaxis followingrenal transplantation. Clin Transplant. 2012;26:E184–190. 10.1111/j.1399-0012.2012.01624.x 22487221

[31] Zmarlicka M, Martin ST, Cardwell SM, Nailor MD. Tolerability of low-dose s ulfamethoxazole /trimethoprim for Pneumocystis jirovecii pneumonia prophylaxis in kidneytransplant recipients. Prog Transplant. 2015;25:210–216. 10.7182/pit2015153 26308779

[32] Kimura M, Tanaka S, Ishikawa A, Endo H, Hirohata S, Kondo H. Comparison of trimethoprim-sulfamethoxazole and aerosolized pentamidine for primary prophylaxis of Pneumocystis jiroveci pneumonia in immunocompromised patients with connective tissuedisease. Rheumatol Int. 2008;28:673–676. 10.1007/s00296-007-0505-4 18080124

[33] Sangiolo D, Storer B, Nash R, Corey L, Davis C, Flowers M, et al. Toxicity and efficacy of daily dapsone as Pneumocystis jiroveci prophylaxis after hematopoietic stem celltransplantation: a case-control study. Biol Blood Marrow Transplant. 2005;11:521–529. 10.1016/j.bbmt.2005.04.011 15983552

[34] Kitazawa T, Seo K, Yoshino Y, Asako K, Kikuchi H, Kono H, et al. Efficacies of atovaquone, pentamidine, and trimethoprim/sulfamethoxazole for the prevention of Pneumocystis jirovecii pneumonia in patients with connective tissue diseases. J Infect Chemother. 2019;25:351–354. 10.1016/j.jiac.2019.01.005 30711257

[35] Hughes W, Shirley K, Subhash C, Sandor F, Manuel V, Rhonies A, et al. Successful chemoprophylaxis for pneumocystis carinii pneumonitis (PCP). The New England Journal of Medicine. 1977;297(26):1419–1426. 10.1056/NEJM197712292972602 412099

