# Supplementary material for: Efficacy and safety of trimethoprim-sulfamethoxazole for the prevention of pneumocystis pneumonia in human immunodeficiency virus-negative immunodeficient patients: A systematic review and meta-analysis
Source: PLoS One. 2021 Mar 25;16(3):e0248524. doi: 10.1371/journal.pone.0248524 (PMC7993619; doi:10.1371/journal.pone.0248524)
Supplement: S1 Table — (DOC) [file pone.0248524.s002.doc]

**S1 Table. Search strategy.**

| **PubMed:** 1203 records (up to December 24, 2020)  (Pneumocystis Pneumonia OR Pneumocystis Infections OR Pneumocystis jirovecii[MeSH Terms]) AND ([Prophylactic](https://www.ncbi.nlm.nih.gov/pubmed/29092853) OR prophylaxis OR prevention[MeSH Terms]) NOT (HIV OR AIDS OR acquired immunodeficiency syndrome OR human immunodeficiency virus[MeSH Terms]) |
| --- |
| **EMBASE:** 669 records (up to December 24, 2020)  ('pneumocystis pneumonia':ti,ab,kw OR 'pneumocystis infections':ti,ab,kw OR 'pneumocystis jirovecii':ti,ab,kw) AND ('prophylactic':ti,ab,kw OR prophylaxis':ti,ab,kw OR 'prevention':ti,ab,kw) NOT ('hiv':ti,ab,kw OR aids':ti,ab,kw OR acquired immunodeficiency syndrome':ti,ab,kw OR 'human immunodeficiency virus':ti,ab,kw) |
| **Web of Science:** 407 records (up to December 24, 2020)  TS=("pneumocystis pneumonia" OR "pneumocystis infections" OR "pneumocystis jirovecii") AND TS=(prophylactic OR prophylaxis OR prevention) NOT TS=(hiv OR aids OR "acquired immunodeficiency syndrome" OR "human immunodeficiency virus") |
| **Cochrane Library:** 113 records (up to December 24, 2020)  (Pneumocystis Pneumonia OR Pneumocystis Infections OR Pneumocystis jirovecii):ti,ab,kw AND (Prophylactic OR prophylaxis OR prevention):ti,ab,kw NOT (HIV OR AIDS OR acquired immunodeficiency syndrome OR human immunodeficiency virus):ti,ab,kw |
