# Supplementary material for: Efficacy and safety of trimethoprim-sulfamethoxazole for the prevention of pneumocystis pneumonia in human immunodeficiency virus-negative immunodeficient patients: A systematic review and meta-analysis
Source: PLoS One. 2021 Mar 25;16(3):e0248524. doi: 10.1371/journal.pone.0248524 (PMC7993619; doi:10.1371/journal.pone.0248524)
Supplement: S2 Table — (DOC) [file pone.0248524.s003.doc]

**S2 Table.** Assessment of the quality of evidence in each included study with the

modified JADAD score .

| **Study** | **Random**  **allocation** | **Concealment**  **schemes** | **Blinding** | **Drop-**  **out** | **integrity of**  **the results** | **Selective**  **report** | **Jadad scale** |
| --- | --- | --- | --- | --- | --- | --- | --- |
| Park 2017  Schmajuk 2018  Sangiolo 2005  Neofytos 2018  Evans 2015  Nazir 2017  Redjoul 2018  Gabardi 2012  Katsuyama 2014  Zmarlicka 2015  Kimura 2008  Ogawa 2005  Okada 1999  Colby 1999  Levinsen 2011  Vananuvat 2011  Kitazawa 2019  Hughes 1977  Ward 1993 | NO  UA  UA  UA  UA  UA  UA  UA  UA  UA  UA  UA  UA  YES  YES  YES  UA  YES  YES | UA  UA  UA  UA  UA  UA  UA  UA  UA  UA  UA  UA  UA  UA  UA  UA  UA  UA  UA | UA  UA  UA  UA  UA  S-B  UA  UA  UA  UA  UA  UA  UA   1. B   UA  UA  S-B  D-B  D-B | Yes  UA  UA  UA  Yes  Yes  Yes  Yes  Yes  Yes  Yes  Yes  Yes  Yes  Yes  Yes  Yes  UA  Yes | Yes  Yes  Yes  Yes  Yes  Yes  Yes  Yes  Yes  Yes  Yes  Yes  Yes  Yes  Yes  Yes  Yes  Yes  Yes | UA  UA  UA  UA  UA  UA  UA  UA  UA  UA  UA  UA  UA  UA  UA  UA  UA  UA  UA | 3  3  3  3  4  4  4  4  4  4  4  4  4  4  5  5  5  5  6 |

Abbreviations: UA:Unclear; S-B:single-blinded; D-B: double-blind; U-B:unblinded.

**Jadad scale:** Points were determined as follows, I. generation of allocation sequence (computer-generated random numbers, 2 points; not described, 1 point; inappropriate method, 0 point); II. allocation concealment (central randomization, sealed envelopes or similar, 2 points; not described, 1 point; inappropriate or unused, 0 point); III. blindness (identical placebo tablets or similar, 2 point; inadequate or not described, 1 point; inappropriate or no double blinding, 0 point); IV. withdrawals and drop-outs (numbers and reasons are described, 1 point; not described, 0 point). The Jadad scale score ranges from 1 to 7; higher score indicates better RCT quality. If a study had a modified Jadad score >4 points, it was considered to be of high quality; if the score was 3-4 points, it was of moderate quality; and if the score was <3 points, it was of low quality.
