# Supplementary material for: Efficacy and safety of trimethoprim-sulfamethoxazole for the prevention of pneumocystis pneumonia in human immunodeficiency virus-negative immunodeficient patients: A systematic review and meta-analysis
Source: PLoS One. 2021 Mar 25;16(3):e0248524. doi: 10.1371/journal.pone.0248524 (PMC7993619; doi:10.1371/journal.pone.0248524)
Supplement: S3 Table — (DOC) [file pone.0248524.s004.doc]

**S3 Table.** Egger's tests for the three Begg's funnel plots (S2 Fig)

| **Std_Eff** | **Coef.** | **Std. Err.** | **t** | **p>|t|** | **[95% Conf. Interval]** |
| --- | --- | --- | --- | --- | --- |
| Egger's test for Fig. S2A. p=0.091>0.05 | | | | | |
| slope  bias | 2.316334  -2.753991 | 1.867912  1.485064 | 1.24  -1.85 | 0.241  0.091 | -1.794912 6.42758 |
|  |  |  |  |  | -6.022594 0.5146129 |
| Egger's test for Fig. S2B. p=0.246>0.05 | | | | | |
| slope  bias | 1.012541  1.914983 | 1.02706  1.330127 | 0.99  1.44 | 0.397  0.246 | -2.256023 4.281105 |
|  |  |  |  |  | -2.318075 6.148041 |
| Egger's test for Fig. S2C. p=0.832>0.05 | | | | | |
| slope  bias | -0.0046401  -0.41552 | 2.145689  1.795071 | -0.00  -0.23 | 0.998  0.832 | -6.833179 6.823899 |
|  |  |  |  |  | -6.128238 5.297198 |
