# Supplementary material for: Efficacy and safety of trimethoprim-sulfamethoxazole for the prevention of pneumocystis pneumonia in human immunodeficiency virus-negative immunodeficient patients: A systematic review and meta-analysis
Source: PLoS One. 2021 Mar 25;16(3):e0248524. doi: 10.1371/journal.pone.0248524 (PMC7993619; doi:10.1371/journal.pone.0248524)
Supplement: S4 Table — (DOC) [file pone.0248524.s005.doc]

**S4 Table.** Sensitivity analyses of the incidence of PCP and rate of drug discontinuation.

| **Outcomes** | **Included Studies** | **Age** | | **Follow-up Duration** | | **Research Type** | |
| --- | --- | --- | --- | --- | --- | --- | --- |
|  |  | **p** | **I^2^** | **p** | **I^2^** | **p** | **I^2^** |
| PCP incidence  Rate of drug discontinuation | 13  5 | 0.01  0.09 | 54%  50% | 0.01  0.09 | 54%  50% | 0.01  0.09 | 54%  50% |
