# Supplementary material for: Efficacy and safety of trimethoprim-sulfamethoxazole for the prevention of pneumocystis pneumonia in human immunodeficiency virus-negative immunodeficient patients: A systematic review and meta-analysis
Source: PLoS One. 2021 Mar 25;16(3):e0248524. doi: 10.1371/journal.pone.0248524 (PMC7993619; doi:10.1371/journal.pone.0248524)
Supplement: S5 Table — (DOC) [file pone.0248524.s006.doc]

**S5 Table.** Heterogeneity analysis for the incidence of PCP and rate of drug discontinuation.

| **Outcomes** | **Excluded Study** | **p** | **I^2^** |
| --- | --- | --- | --- |
| PCP incidence  Rate of drug discontinuation | Hughes 1977  Katsuyama 2014  Levinsen 2011  Neofytos 2018  Ogawa 2005  Okada 1999  Park 2017  Ward 1993  Vananuvat 2011  Kimura 2008  Nazir 2017  Redjoul 2018  Sangiolo 2005  Gabardi 2012  Kitazawa 2019  Nazir 2017  Colby 1999  Redjoul 2018 | 0.02  0.006  0.008  0.51  0.007  0.006  0.01  0.01  0.008  0.009  0.009  0.008  0.007    0.05  0.92  0.13  0.06  0.10 | 50%  58%  57%  0%  58%  58%  55%  55%  57%  56%  56%  56%  57%  62%  0%  46%  60%  52% |
