# Supplementary figures and images for: Efficacy and safety of trimethoprim-sulfamethoxazole for the prevention of pneumocystis pneumonia in human immunodeficiency virus-negative immunodeficient patients: A systematic review and meta-analysis
Source: PLoS One. 2021 Mar 25;16(3):e0248524. doi: 10.1371/journal.pone.0248524 (PMC7993619; doi:10.1371/journal.pone.0248524)

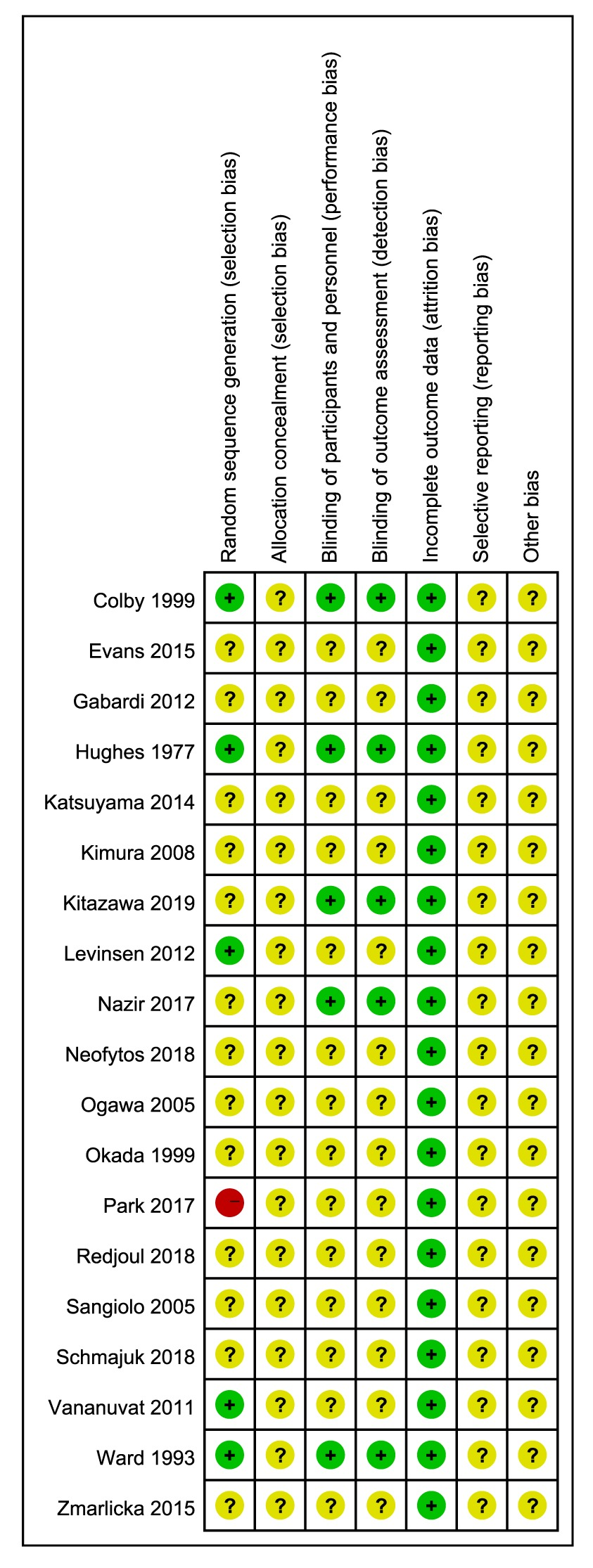

Supplement: S1 Fig — Each risk of bias item for each included study was reviewed. (TIF) [file pone.0248524.s007.tif]

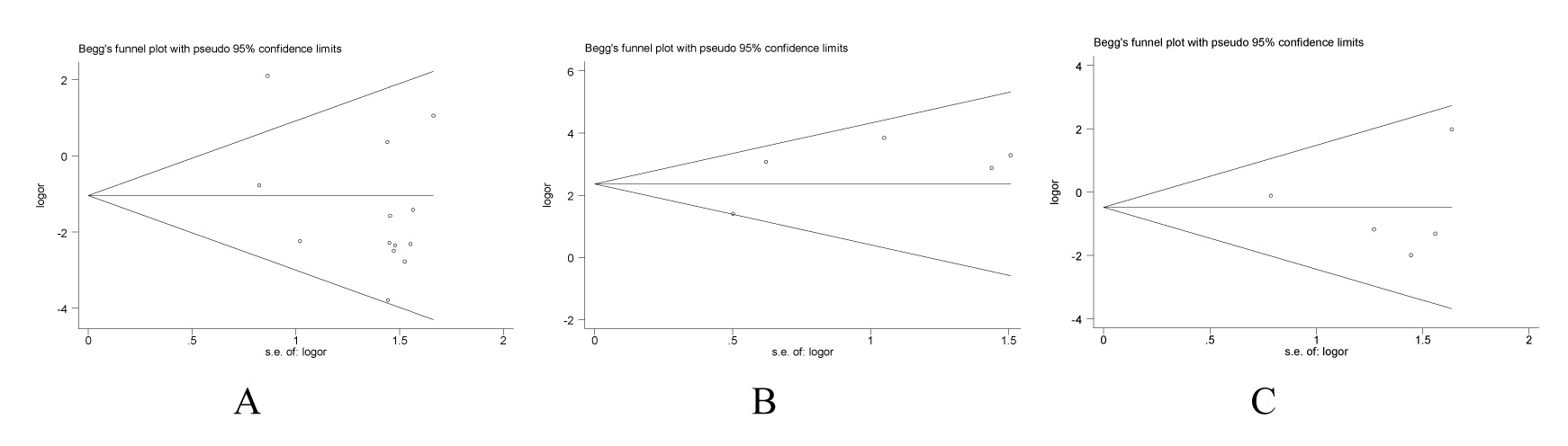

Supplement: S2 Fig — Begg’s funnel plots for the meta-analysis of the incidence of PCP (A), rate of drug discontinuation (B) and rate of mortality (C). (TIF) [file pone.0248524.s008.tif]
